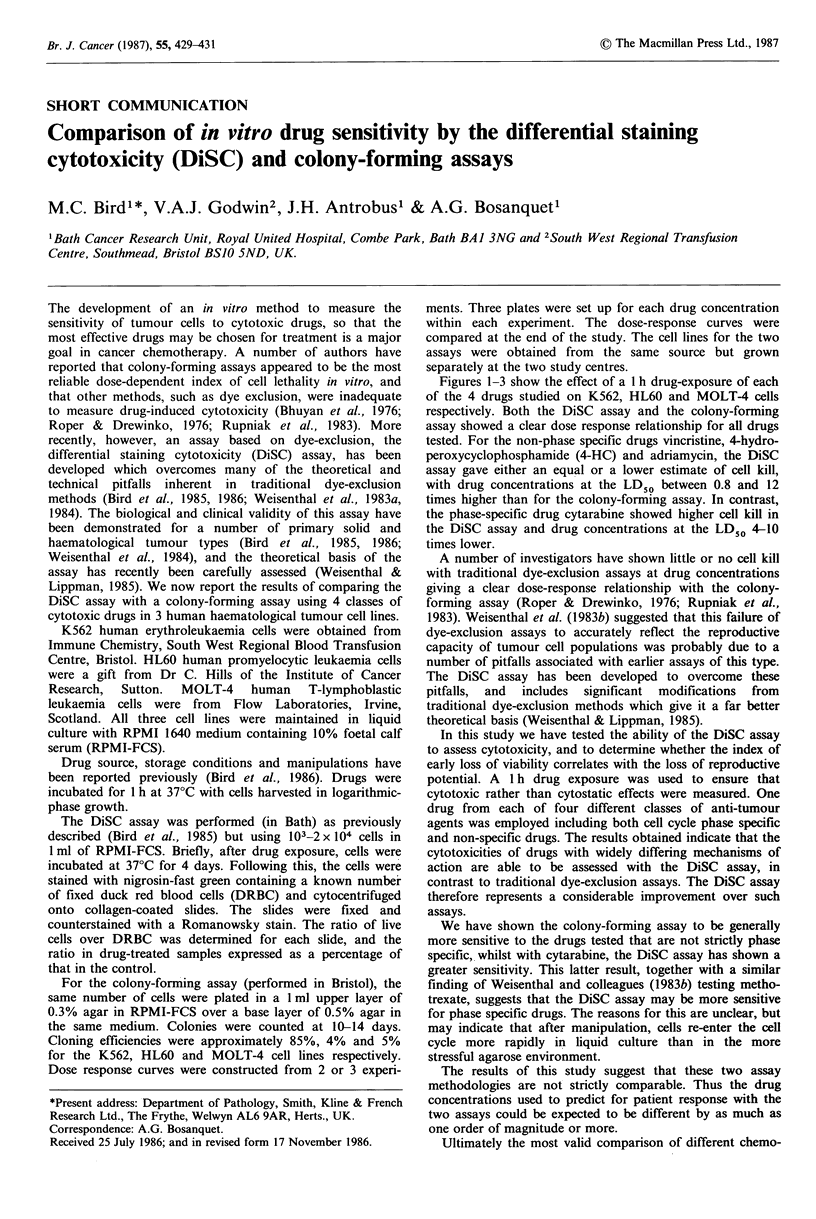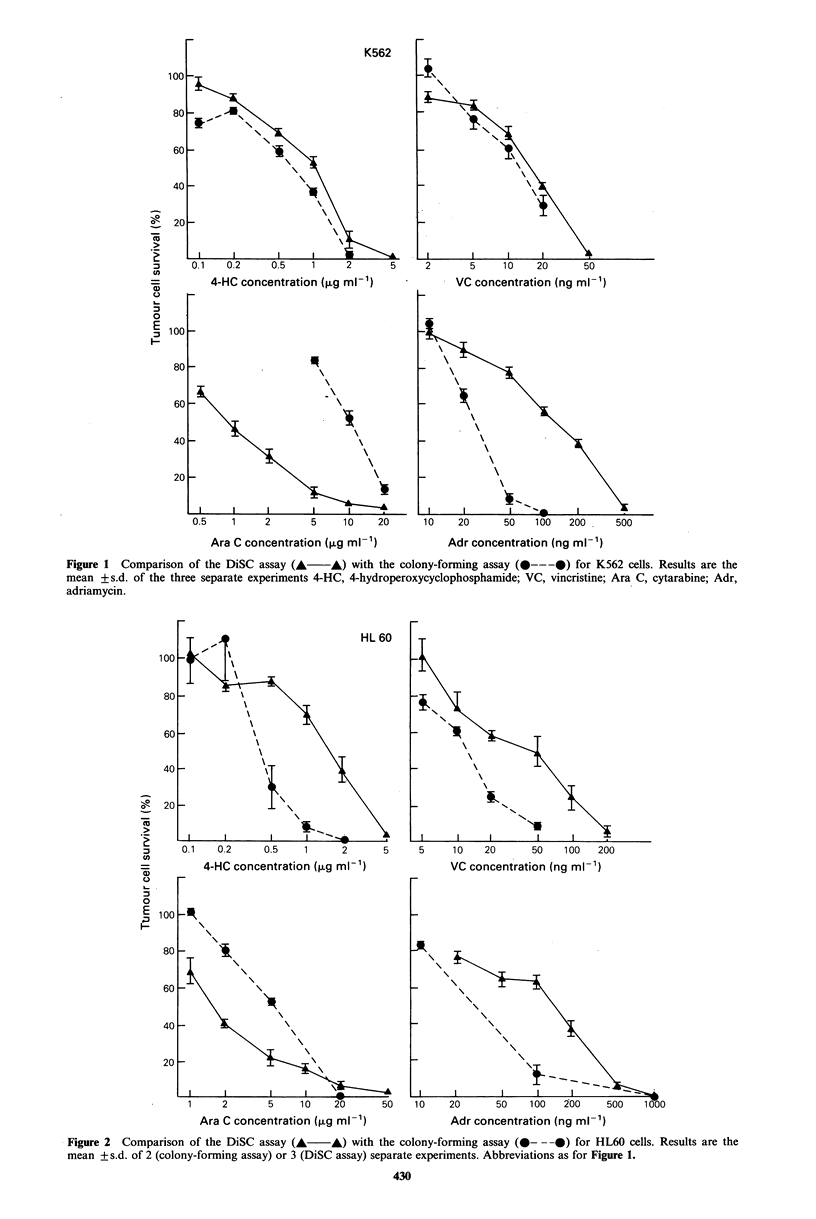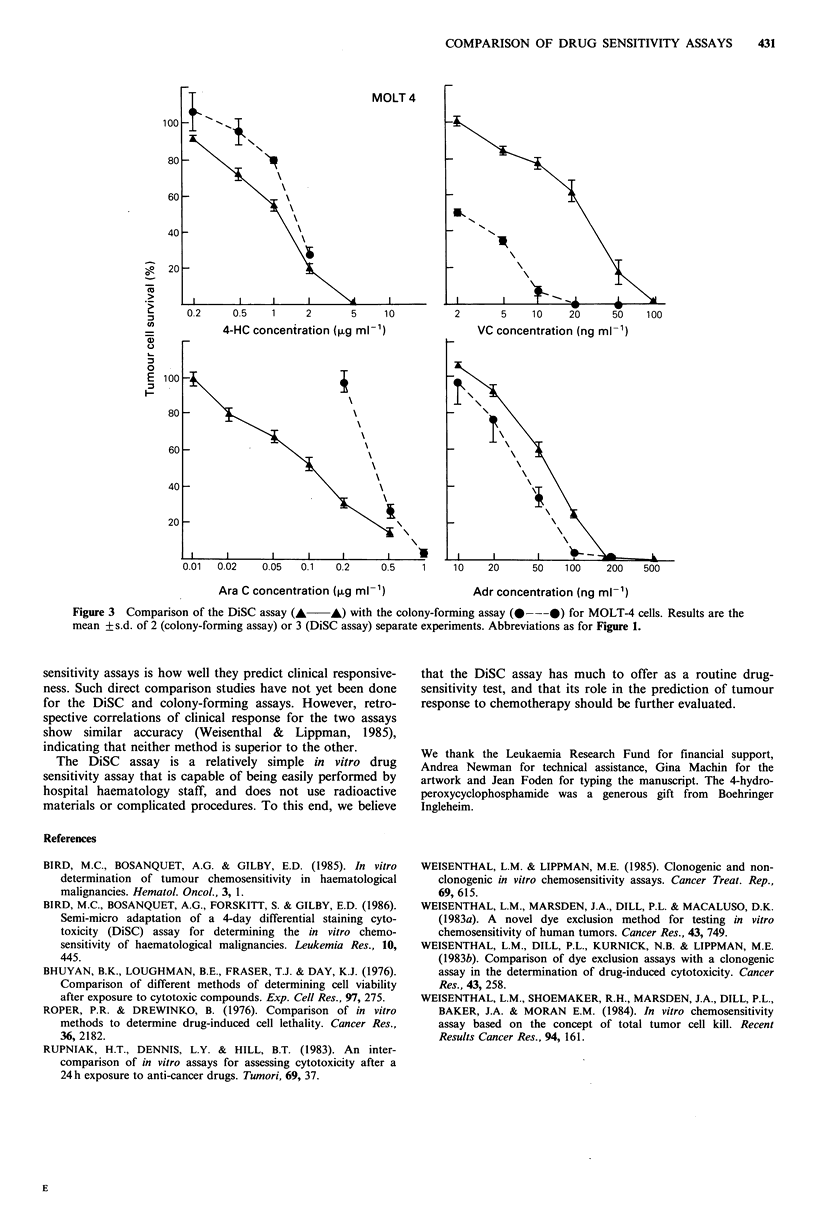# Comparison of in vitro drug sensitivity by the differential staining cytotoxicity (DiSC) and colony-forming assays.

**DOI:** 10.1038/bjc.1987.84

**Published:** 1987-04

**Authors:** M. C. Bird, V. A. Godwin, J. H. Antrobus, A. G. Bosanquet


*Br. J. Cancer* (1987), **55**, 429–431 © The Macmillan Press Ltd., 1987

SHORT COMMUNICATION

### Comparison of *in vitro* drug sensitivity by the differential staining cytotoxicity (DiSC) and colony-forming assays

M.C. Bird[1]*, V.A.J. Godwin[2], J.H. Antrobus[1] & A.G. Bosanquet[1]

[1]*Bath Cancer Research Unit, Royal United Hospital, Combe Park, Bath BA1 3NG and* [2]*South West Regional Transfusion Centre, Southmead, Bristol BS10 5ND, UK.*

The development of an *in vitro* method to measure the sensitivity of tumour cells to cytotoxic drugs, so that the most effective drugs may be chosen for treatment is a major goal in cancer chemotherapy. A number of authors have reported that colony-forming assays appeared to be the most reliable dose-dependent index of cell lethality *in vitro*, and that other methods, such as dye exclusion, were inadequate to measure drug-induced cytotoxicity (Bhuyan *et al.*, 1976; Roper & Drewinko, 1976; Rupniak *et al.*, 1983). More recently, however, an assay based on dye-exclusion, the differential staining cytotoxicity (DiSC) assay, has been developed which overcomes many of the theoretical and technical pitfalls inherent in traditional dye-exclusion methods (Bird *et al.*, 1985, 1986; Weisenthal *et al.*, 1983*a*, 1984). The biological and clinical validity of this assay have been demonstrated for a number of primary solid and haematological tumour types (Bird *et al.*, 1985, 1986; Weisenthal *et al.*, 1984), and the theoretical basis of the assay has recently been carefully assessed (Weisenthal & Lippman, 1985). We now report the results of comparing the DiSC assay with a colony-forming assay using 4 classes of cytotoxic drugs in 3 human haematological tumour cell lines.

K562 human erythroleukaemia cells were obtained from Immune Chemistry, South West Regional Blood Transfusion Centre, Bristol. HL60 human promyelocytic leukaemia cells were a gift from Dr C. Hills of the Institute of Cancer Research, Sutton. MOLT-4 human T-lymphoblastic leukaemia cells were from Flow Laboratories, Irvine, Scotland. All three cell lines were maintained in liquid culture with RPMI 1640 medium containing 10% foetal calf serum (RPMI-FCS).

Drug source, storage conditions and manipulations have been reported previously (Bird *et al.*, 1986). Drugs were incubated for 1 h at 37°C with cells harvested in logarithmic-phase growth.

The DiSC assay was performed (in Bath) as previously described (Bird *et al.*, 1985) but using $10^3$–$2\times10^4$ cells in 1 ml of RPMI-FCS. Briefly, after drug exposure, cells were incubated at 37°C for 4 days. Following this, the cells were stained with nigrosin-fast green containing a known number of fixed duck red blood cells (DRBC) and cytocentrifuged onto collagen-coated slides. The slides were fixed and counterstained with a Romanowsky stain. The ratio of live cells over DRBC was determined for each slide, and the ratio in drug-treated samples expressed as a percentage of that in the control.

For the colony-forming assay (performed in Bristol), the same number of cells were plated in a 1 ml upper layer of 0.3% agar in RPMI-FCS over a base layer of 0.5% agar in the same medium. Colonies were counted at 10–14 days. Cloning efficiencies were approximately 85%, 4% and 5% for the K562, HL60 and MOLT-4 cell lines respectively. Dose response curves were constructed from 2 or 3 experiments. Three plates were set up for each drug concentration within each experiment. The dose-response curves were compared at the end of the study. The cell lines for the two assays were obtained from the same source but grown separately at the two study centres.

Figures 1–3 show the effect of a 1 h drug-exposure of each of the 4 drugs studied on K562, HL60 and MOLT-4 cells respectively. Both the DiSC assay and the colony-forming assay showed a clear dose response relationship for all drugs tested. For the non-phase specific drugs vincristine, 4-hydroperoxycyclophosphamide (4-HC) and adriamycin, the DiSC assay gave either an equal or a lower estimate of cell kill, with drug concentrations at the $LD_{50}$ between 0.8 and 12 times higher than for the colony-forming assay. In contrast, the phase-specific drug cytarabine showed higher cell kill in the DiSC assay and drug concentrations at the $LD_{50}$ 4–10 times lower.

A number of investigators have shown little or no cell kill with traditional dye-exclusion assays at drug concentrations giving a clear dose-response relationship with the colony-forming assay (Roper & Drewinko, 1976; Rupniak *et al.*, 1983). Weisenthal *et al.* (1983*b*) suggested that this failure of dye-exclusion assays to accurately reflect the reproductive capacity of tumour cell populations was probably due to a number of pitfalls associated with earlier assays of this type. The DiSC assay has been developed to overcome these pitfalls, and includes significant modifications from traditional dye-exclusion methods which give it a far better theoretical basis (Weisenthal & Lippman, 1985).

In this study we have tested the ability of the DiSC assay to assess cytotoxicity, and to determine whether the index of early loss of viability correlates with the loss of reproductive potential. A 1 h drug exposure was used to ensure that cytotoxic rather than cytostatic effects were measured. One drug from each of four different classes of anti-tumour agents was employed including both cell cycle phase specific and non-specific drugs. The results obtained indicate that the cytotoxicities of drugs with widely differing mechanisms of action are able to be assessed with the DiSC assay, in contrast to traditional dye-exclusion assays. The DiSC assay therefore represents a considerable improvement over such assays.

We have shown the colony-forming assay to be generally more sensitive to the drugs tested that are not strictly phase specific, whilst with cytarabine, the DiSC assay has shown a greater sensitivity. This latter result, together with a similar finding of Weisenthal and colleagues (1983*b*) testing methotrexate, suggests that the DiSC assay may be more sensitive for phase specific drugs. The reasons for this are unclear, but may indicate that after manipulation, cells re-enter the cell cycle more rapidly in liquid culture than in the more stressful agarose environment.

The results of this study suggest that these two assay methodologies are not strictly comparable. Thus the drug concentrations used to predict for patient response with the two assays could be expected to be different by as much as one order of magnitude or more.

Ultimately the most valid comparison of different chemo-

*Present address: Department of Pathology, Smith, Kline & French Research Ltd., The Frythe, Welwyn AL6 9AR, Herts., UK.
Correspondence: A.G. Bosanquet.
Received 25 July 1986; and in revised form 17 November 1986.

**Figure 1** Comparison of the DiSC assay (▲——▲) with the colony-forming assay (●---●) for K562 cells. Results are the mean ±s.d. of the three separate experiments 4-HC, 4-hydroperoxycyclophosphamide; VC, vincristine; Ara C, cytarabine; Adr, adriamycin.

**Figure 2** Comparison of the DiSC assay (▲——▲) with the colony-forming assay (●---●) for HL60 cells. Results are the mean ±s.d. of 2 (colony-forming assay) or 3 (DiSC assay) separate experiments. Abbreviations as for **Figure 1**.

**Figure 3** Comparison of the DiSC assay (▲——▲) with the colony-forming assay (●---●) for MOLT-4 cells. Results are the mean ±s.d. of 2 (colony-forming assay) or 3 (DiSC assay) separate experiments. Abbreviations as for **Figure 1**.

sensitivity assays is how well they predict clinical responsiveness. Such direct comparison studies have not yet been done for the DiSC and colony-forming assays. However, retrospective correlations of clinical response for the two assays show similar accuracy (Weisenthal & Lippman, 1985), indicating that neither method is superior to the other.

The DiSC assay is a relatively simple *in vitro* drug sensitivity assay that is capable of being easily performed by hospital haematology staff, and does not use radioactive materials or complicated procedures. To this end, we believe that the DiSC assay has much to offer as a routine drug-sensitivity test, and that its role in the prediction of tumour response to chemotherapy should be further evaluated.

We thank the Leukaemia Research Fund for financial support, Andrea Newman for technical assistance, Gina Machin for the artwork and Jean Foden for typing the manuscript. The 4-hydroperoxycyclophosphamide was a generous gift from Boehringer Ingleheim.

**References**

BIRD, M.C., BOSANQUET, A.G. & GILBY, E.D. (1985). *In vitro* determination of tumour chemosensitivity in haematological malignancies. *Hematol. Oncol.*, **3**, 1.

BIRD, M.C., BOSANQUET, A.G., FORSKITT, S. & GILBY, E.D. (1986). Semi-micro adaptation of a 4-day differential staining cytotoxicity (DiSC) assay for determining the *in vitro* chemosensitivity of haematological malignancies. *Leukemia Res.*, **10**, 445.

BHUYAN, B.K., LOUGHMAN, B.E., FRASER, T.J. & DAY, K.J. (1976). Comparison of different methods of determining cell viability after exposure to cytotoxic compounds. *Exp. Cell Res.*, **97**, 275.

ROPER, P.R. & DREWINKO, B. (1976). Comparison of *in vitro* methods to determine drug-induced cell lethality. *Cancer Res.*, **36**, 2182.

RUPNIAK, H.T., DENNIS, L.Y. & HILL, B.T. (1983). An intercomparison of *in vitro* assays for assessing cytotoxicity after a 24 h exposure to anti-cancer drugs. *Tumori*, **69**, 37.

WEISENTHAL, L.M. & LIPPMAN, M.E. (1985). Clonogenic and nonclonogenic *in vitro* chemosensitivity assays. *Cancer Treat. Rep.*, **69**, 615.

WEISENTHAL, L.M., MARSDEN, J.A., DILL, P.L. & MACALUSO, D.K. (1983*a*). A novel dye exclusion method for testing *in vitro* chemosensitivity of human tumors. *Cancer Res.*, **43**, 749.

WEISENTHAL, L.M., DILL, P.L., KURNICK, N.B. & LIPPMAN, M.E. (1983*b*). Comparison of dye exclusion assays with a clonogenic assay in the determination of drug-induced cytotoxicity. *Cancer Res.*, **43**, 258.

WEISENTHAL, L.M., SHOEMAKER, R.H., MARSDEN, J.A., DILL, P.L., BAKER, J.A. & MORAN E.M. (1984). *In vitro* chemosensitivity assay based on the concept of total tumor cell kill. *Recent Results Cancer Res.*, **94**, 161.